# Distribution patterns of metastatic pelvic lymph nodes assessed by CT/MRI in patients with uterine cervical cancer

**DOI:** 10.1186/1748-717X-8-139

**Published:** 2013-06-08

**Authors:** Goro Kasuya, Takafumi Toita, Kazuhisa Furutani, Takeshi Kodaira, Tatsuya Ohno, Yuko Kaneyasu, Ryouichi Yoshimura, Takashi Uno, Akira Yogi, Satoshi Ishikura, Masahiro Hiraoka

**Affiliations:** 1 Department of Radiology, Graduate School of Medical Science, University of the Ryukyus, Okinawa, Japan; 2 Department of Radiation Oncology, Aichi Cancer Center, Nagoya, Japan; 3 Heavy Ion Medical Center, Gunma University, Maebashi, Japan; 4 Department of Radiation Oncology, Graduate School of Biomedical Sciences, Hiroshima University, Hiroshima, Japan; 5 Department of Radiology, Tokyo Medical and Dental University, Tokyo, Japan; 6 Department of Radiology, Graduate School of Medicine, Chiba University, Chiba, Japan; 7 Department of Radiation Oncology, Juntendo University, Tokyo, Japan; 8 Department of Radiation Oncology and Image-applied Therapy, Kyoto University Graduate School of Medicine, Kyoto, Japan

**Keywords:** Radiotherapy, Lymph node, Clinical target volume, Uterine cervical cancer

## Abstract

**Background:**

To investigate the three-dimensional (3D) distribution patterns of clinically metastatic (positive) lymph nodes on pretreatment computed tomography (CT)/magnetic resonance imaging (MRI) images of patients with locally advanced cervical cancer.

**Methods:**

We enrolled 114 patients with uterine cervical cancer with positive nodes by CT/MRI (≥10 mm in the shortest diameter). Pretreatment CT/MRI data were collected at 6 institutions. The FIGO stage was IB1 in 2 patients (2%), IB2 in 6 (5%), IIA in 3 (3%), IIB in 49 (43%), IIIB in 50 (44%), and IVA in 4 (4%) patients. The median cervical tumor diameter assessed by T2-weighted MRI was 55 mm (range, 10–87 mm). The anatomical distribution of the positive nodes was evaluated on CT/MRI images by two radiation oncologists and one diagnostic radiologist.

**Results:**

In these patients, 273 enlarged nodes were assessed as positive. The incidence of positive nodes was 104/114 (91%) for the obturator region, 31/114 (27%) for the external iliac region, 16/114 (14%) for the internal iliac region, 22/114 (19%) for the common iliac region, and 6/114 (5%) for the presacral region. The external iliac region was subdivided into four sub-regions: lateral, intermediate, medial, and caudal. The obturator region was subdivided into two sub-regions: cranial and caudal. The majority of patients had positive nodes in the cranial obturator and/or the medial external iliac region (111/114). In contrast, few had positive nodes in the lateral external iliac, caudal external iliac, caudal obturator, internal iliac and presacral regions. All cases with positive nodes in those low-risk regions also had positive nodes in other pelvic nodal regions concomitantly. The incidence of positive nodes in the low-risk regions/sub-regions was significantly related to FIGO stage (p=0.017) and number of positive nodes (p<0.001).

**Conclusions:**

We demonstrated the 3D distribution patterns of clinical metastatic pelvic lymph nodes on pretreatment CT/MRI images of patients with locally advanced cervical cancer. These findings might contribute to future individualization of the clinical target volume of the pelvic nodes in patients with cervical cancer.

## Background

Radiotherapy plays very important roles in the treatment of uterine cervical cancer. Definitive radiotherapy for cervical cancer consists of external beam radiotherapy and intracavitary brachytherapy. Recently, external beam radiotherapy techniques have advanced considerably, as have those for intracavitary brachytherapy. Treatment planning for uterine cervical cancer has transitioned from a two-dimensional (2D) approach based on bony landmarks to a three-dimensional (3D) technique based on computed tomography (CT)/magnetic resonance imaging (MRI). Intensity-modulated radiotherapy (IMRT) has been proven to have a significant dosimetric advantage and less toxicity compared with conventional 2D/3D treatment planning for various malignancies, including gynecologic cancers [1]. It is essential to define the proper clinical target volume (CTV) for appropriate delivery of IMRT. Guidelines that provide a standard definition of CTV nodes are now published by the Radiation Therapy Oncology Group (RTOG) [2], UK investigators [3] and the Japan Clinical Oncology Group (JCOG) [4]. However, these guidelines were developed mainly from information on the normal anatomical pelvic lymph node distribution. The actual distribution of clinically metastatic (positive) nodes in the pelvis has not been studied in definitive radiotherapy series. If areas with a low risk of node metastases could be deleted from the CTV, toxicity could be reduced without sacrificing regional control.

The purpose of this study was to investigate the 3D distribution patterns of clinically metastatic nodes assessed by CT/MRI in patients with uterine cervical cancer.

## Methods

We enrolled 114 patients with uterine cervical cancer who were diagnosed as having clinically metastatic (positive) pelvic nodes by CT/MRI (≥10 mm in the shortest diameter) and treated by definitive radiotherapy/chemoradiotherapy at 6 institutions between January 2001 and December 2007. This study conformed to the ethical principles contained in the Declaration of Helsinki [5], and was approved by the institutional review board of the principal investigator (T.T.). Lymph nodes greater than or equal to 10 mm in the shortest diameter, as assessed by CT/MRI, were defined as positive in this study. Patient characteristics are summarized in Table 1. Digitized CT/MRI images burned to CD-ROMs were collected from each institution. The images were reviewed by two radiation oncologists (G.K., T.T.) and one diagnostic radiologist (A.Y.).

**Table 1:** Patient characteristics (n=114)

**Characteristic**	**(n)**
FIGO stage	
IB1	2
IB2	6
IIA	3
IIB	49
IIIB	50
IVA	4
Age	
median 52	range 26-88
Histology	
SCC	109
Adeno	5
Tumor size*	
<20 mm	0
21-40 mm	18
41-60 mm	58
61 mm<	38
Number of metastatic LN in the pelvis	
1	32
2	36
3	23
4	14
≥5	9

*assessed by MRI.

FIGO=Federation Internationale de Gynecologie et de Obstetrique.

SCC=Squamous cell carcinoma.

LN=Lymph node.

**Figure 1:**
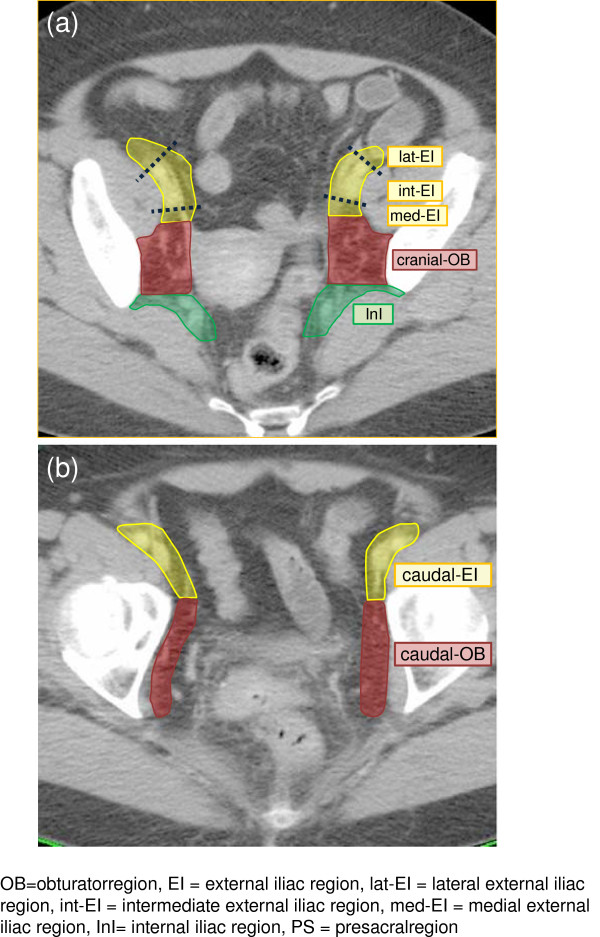
**Atlas of the CTV nodes: regions and sub-regions.** Middle-level of pelvis' for (**a**), and 'low-level of pelvis' for (**b**).

Pelvic lymph node area was divided into five anatomical regions: the obturator region, the external iliac region, the internal iliac region, the common iliac region, and the presacral region. The external iliac was further divided into four sub-regions: the medial external iliac, the intermediate external iliac, the lateral external iliac, and the caudal external iliac. The subcategories of medial external iliac, intermediate external iliac, and lateral external iliac refer to the definitions proposed by Taylor et al. [6] and Lengelé et al. [7]: medial external iliac=the dorsal area of attachment and along the external iliac vein, intermediate external iliac=the anterior area between the external iliac artery and vein, and lateral external iliac=the lateral area of the external iliac artery. These three sub-regions are all located cranial to the aspect of the femoral head. On the other hand, the caudal external iliac is located caudal to the aspect of the femoral head. The obturator was also divided into two sub-regions, with the border of the aspect of the femoral head as the external iliac: cranial obturator and caudal obturator. An atlas of these sub-regions (except for common iliac region) is presented in Figure 1 (a)-(b).

First, the number of positive nodes in each region and in the sub-regions was counted. Next, the distribution patterns of the positive nodes were analyzed in each area.

Statistical analyses were performed with the chi-square test. A probability level of 0.05 was chosen for statistical significance.

## Results

**Figure 2:**
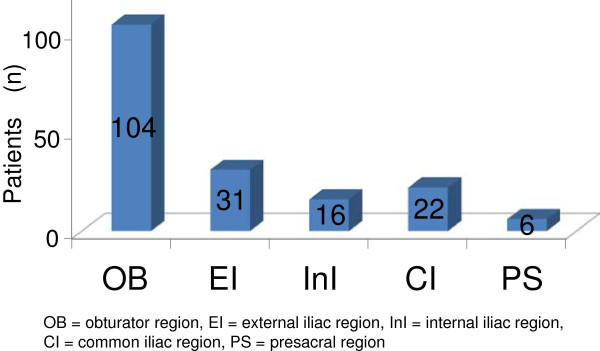
**Incidence of clinically metastatic pelvic lymph nodes in each region.** *Including duplication.

There were 273 positive nodes as assessed by CT/MRI. The median number of positive nodes per patient was 2 (range, 1–7). Figure 2 shows the incidence of positive nodes in each nodal region. The area that most frequently contained positive nodes was the obturator region. In contrast, positive nodes were rarely observed in the presacral region. Table 2 shows the anatomical distribution of positive nodes in the pelvis. A solitary positive node was observed only in the obturator and the external iliac regions. In contrast, no solitary positive node was observed in the internal iliac, common iliac, and presacral regions. Within the obturator and external iliac regions, positive nodes were rarely observed in the caudal and lateral external iliac sub-regions. Ninety-seven percent of the patients (111/114) had one or more positive nodes in the cranial obturator and/or the medial external iliac regions. A solitary positive node was observed only in the cranial obturator, and medial/intermediate external iliac regions. For other regions or sub-regions, patients with positive nodes also had positive nodes concomitantly in other pelvic nodal regions or sub-regions. We defined each of these regions as a NSR (non-solitary region). Patients with NSR metastases had high FIGO stages or a large number of positive nodes. Patients with high FIGO-stage disease (III, IVA) had a significantly higher frequency of positive nodes in the NSR (23/54, 43%) compared with patients who had low FIGO-stage disease (15/60, 25%) (P=0.047). The average number of metastatic lymph nodes was 3.7 for patients with NSR and 1.9 for patients with non-NSR. There was no significant relationship between tumor diameter and the incidence of NSR metastases: 6/18 (30%) for tumors≤40 mm, 20/58 (34%) for tumors 41–60 mm, and 15/38 (38%) for tumors≥61 mm. All 16 patients with nodal metastases in less common (≤ 6/114, 5%) areas (presacral, caudal obturator, and caudal/lateral external iliac regions) had large tumors (> 4 cm), of which all 9 cases who had NSR metastases in the lateral external iliac and presacral regions were stage IIB or more. In addition, all 9 cases with NSR metastases in the caudal obturator or caudal external iliac regions also had positive lymph nodes in the ipsilateral cranial obturator or medial external iliac region, and all 3 cases with NSR metastases in the lateral external iliac region had positive nodes in the ipsilateral intermediate external iliac region.

**Table 2:** Number of patients* with clinically pelvic nodal metastases # by region/subdivided region

	**Total**	**OB**		**EI**				**InI**	**CI**	**PS**
		**Cranial**	**Caudal**	**Med**	**Int**	**Lat**	**Caudal**			
Positive nodes in other regions	82	76	5	10	13	3	6	16	22	6
No positive nodes in other regions	32	28	0	2	2	0	0	0	0	0
Total	114	104	5	12	15	3	6	16	22	6

*including duplication, # assesed by CT/MRI (>= 10 mm in shortest diameter).

OB = obturator region, EI = external iliac region, InI = internal iliac region, CI = common iliac region, PS = presacral region, Med = medial, Int = intermediate, Lat = lateral.

## Discussion

To minimize the risk of inter-planner variability on pelvic node CTV contouring, consensus-based CTV guidelines have been developed for patients with cervical cancer [2-4]. Modification of the standard CTV guidelines based on the probability of subclinical disease, in other words, risk of recurrence is the next challenge for individualized treatment planning.

The CTV could be divided into subgroups, e.g., high-risk CTV and low-risk CTV, according to the probability of recurrence. The high-risk CTV would be defined as the volume that involves frequent metastases, and should be treated for every patient. In contrast, the low-risk CTV would be defined as a region with rare disease involvement, and might be able to be excluded from the CTV in certain situations. The arrangement of CTV nodes could reduce the dose/volume of organs at risk (OAR) and lead to lower side effects. In the previously published guidelines, the CTV nodes cover the entire anatomical pelvic node distribution [2-4,6]. The guidelines did not emphasize the actual probability of nodal involvement, in other words, the risk of recurrence. In head and neck cancers, individualization of CTV nodes for 3D planning was proposed according to the primary site or T/N stage [8]. For uterine cervical cancer, in an attempt at dose reduction for OAR, small pelvic field irradiation has been investigated [9,10]. The treatment fields were designed to exclude the common iliac region. In this study, we tried to analyze the 3D distribution patterns of positive nodes in the pelvis assessed by pretreatment CT/MRI to quantify the nodal metastasis probability in patients with uterine cervical cancer.

Some surgical series have indicated that the obturator and external iliac regions have the highest frequency of metastatic lymph nodes [11,12]. This is consistent with the findings demonstrated in the present study. We subdivided the obturator and external iliac regions according to craniocaudal distribution at the border of the femoral head. Analyses with this subdivision revealed that positive lymph nodes were rarely seen on the caudal side, and most were observed on the cranial side. Benedetti and colleagues also subdivided the obturator region into deep and superficial regions. They demonstrated that there were few metastatic lymph nodes in the deep region [12]. Our results are consistent with that report. No previous study supports our finding that the caudal external iliac sub-region rarely had positive nodes. However, the present results suggest the appropriateness of the definition of the external iliac region in the RTOG and JCOG guidelines, which set the lower end of the external iliac region at the top of the femoral head [2,4].

For the external iliac region above the aspect of the femoral head, 3 anatomically subdivided regions have been proposed [6,7]. According to the definition, positive nodes in the medial external iliac and intermediate external iliac regions were frequent in contrast to the lateral external iliac regions in our study. This observation was also made by Graham et al. [13]. Taylor et al. demonstrated that the normal node distribution extended more than 10 mm laterally to the external iliac artery and veins in their USPIO MRI study. Based on this finding, they recommended that the CTV should expand 17 mm laterally from the vessels to cover the region sufficiently [6]. However, our present study demonstrated that positive nodes were rare in the lateral external iliac region, and suggested that the expansion that Taylor proposed could be omitted in some cases.

Based on the findings from our present analyses, high-risk regions such as the cranial obturator and the medial and intermediate external regions must be irradiated sufficiently in all cases. In contrast, the caudal external iliac, caudal obturator, lateral external iliac and presacral regions, which demonstrated a very low incidence (≤ 5%) of positive nodes, might be allowed to be excluded in patients who satisfy all of the following criteria: small tumor size (≤ 4 cm) and no positive nodes on CT/MRI. The CTV shrinkage might help to reduce complications in the surrounding organs. Further investigation is needed to justify such modification in clinical practice. In the other remaining regions (i.e., common iliac, internal iliac), although no patient in this study had a solitary positive lymph node, the positive rate was not low. Therefore, we suggest that the common iliac and internal iliac regions should continue to be included in all cases for radical radiotherapy for patients with uterine cervical cancer.

This study has some limitations. First, the insufficient sensitivity of CT/MRI is critical. Bellomi and colleagues reported that the sensitivity and specificity of CT were 64% and 93%, respectively, and those of MRI were 72% and 93%, respectively [14]. The results of this study should be interpreted carefully due to the inadequate sensitivity and specificity of these methods. Meanwhile, Choi and coworkers compared the sensitivity and specificity of MRI and positron emission tomography/computed tomography (PET/CT) [15]. With MRI, the sensitivity and specificity were 30% and 92%, respectively, and with PET/CT, the sensitivity and specificity were 57% and 92%, respectively. Further study using PET/CT is encouraged. Second, the absence of histopathological confirmation is a serious weakness of the present study. Although some surgical series presented detailed data on the pathological positive node distribution [11,12], data for inoperable advanced-stage patients were sparse. In addition, it is difficult to apply the distribution of metastatic nodes from surgical findings directly to the 3D distribution on CT/MRI images for accurate CTV contouring. For these reasons, despite its insufficient sensitivity and specificity, some surrogate information could be obtained from the study using CT/MRI. Third, this study consists of a relatively small number of patients and a heterogenous population (i.e. stage, tumor size). Various systematic and random errors due to multicenter assessment over a long time period might negatively affect the validity of the study.

## Conclusions

The present study demonstrated distribution patterns of positive pelvic nodes in patients with cervical cancer treated with definitive radiotherapy/chemoradiotherapy. The findings might contribute to future investigations for the individualization of CTV node contouring.

## Abbreviations

3D: Three-dimensional; CT: Computed tomography; MRI: Magnetic resonance imaging; FIGO: Federation Internationale de Gynecologie et de Obstetrique; RTOG: Radiation Therapy Oncology Group; UK: United Kingdom; JCOG: Japan Clinical Oncology Group; CTV: Clinical target volume; NSR: Non-solitary region; OAR: Organs at risk; PET: Positron emission tomography.

## Competing interests

The authors declare that they have no competing interests regarding this manuscript.

## Authors’ contributions

GK and TT designed this study, assembled the data, performed the statistical analysis and interpretation, and wrote the manuscript. KF, TK, TO, YK, RY, and TU provided study materials from each institution and proofed the manuscript. AY participated as a diagnostic radiologist and confirmed the distribution of positive lymph nodes. SI and MH helped to revise the manuscript. All authors read and approved the final manuscript.
